# Supplementary material for: HIVprotI: an integrated web based platform for prediction and design of HIV proteins inhibitors
Source: J Cheminform. 2018 Mar 9;10:12. doi: 10.1186/s13321-018-0266-y (PMC5845081; doi:10.1186/s13321-018-0266-y)
Supplement: Supplementary file 2 — Additional file 2. Source code of HIVProtI web server. [file 13321_2018_266_MOESM2_ESM.zip › HIVprotI_Source-code/hivprotI/weka-3-6-6/doc/weka/experiment/RemoteExperimentEvent.html]

RemoteExperimentEvent


---


|  |  |  |
| --- | --- | --- |
| **Overview** \| **Package** \| **Class** \| **Tree** \| **Deprecated** \| **Index** \| **Help** \| **Weka's home** | |  |
| **PREV CLASS**   **NEXT CLASS** | **FRAMES**    **NO FRAMES**     **All Classes** |  |
| SUMMARY: NESTED \| FIELD \| CONSTR \| METHOD | DETAIL: FIELD \| CONSTR \| METHOD |  |


---


## weka.experiment Class RemoteExperimentEvent

```
java.lang.Object
  weka.experiment.RemoteExperimentEvent
```

**All Implemented Interfaces:**: java.io.Serializable

---

``` public class RemoteExperimentEvent extends java.lang.Object implements java.io.Serializable ```

Class encapsulating information on progress of a remote experiment

**Version:**
:   $Revision: 1.7 $

**Author:**
:   Mark Hall (mhall@cs.waikato.ac.nz)

**See Also:**: Serialized Form

---

| **Field Summary** | |
| --- | --- |
| `boolean` | `m_experimentFinished`             True if a remote experiment has finished |
| `boolean` | `m_logMessage`             A log type message |
| `java.lang.String` | `m_messageString`             The message |
| `boolean` | `m_statusMessage`             A status type message |


**Constructor Summary**

`RemoteExperimentEvent(boolean status,
boolean log,
boolean finished,
java.lang.String message)`
  
          Constructor


**Method Summary**

**Methods inherited from class java.lang.Object**

`equals, getClass, hashCode, notify, notifyAll, toString, wait, wait, wait`

**Field Detail**

### m\_statusMessage

```
public boolean m_statusMessage
```

:   A status type message

---


### m\_logMessage

```
public boolean m_logMessage
```

:   A log type message

---


### m\_messageString

```
public java.lang.String m_messageString
```

:   The message

---


### m\_experimentFinished

```
public boolean m_experimentFinished
```

:   True if a remote experiment has finished


**Constructor Detail**

### RemoteExperimentEvent

```
public RemoteExperimentEvent(boolean status,
                             boolean log,
                             boolean finished,
                             java.lang.String message)
```

:   Constructor

    **Parameters:**: `status` - true for status type messages: `log` - true for log type messages: `finished` - true if experiment has finished: `message` - the message


---


|  |  |  |
| --- | --- | --- |
| **Overview** \| **Package** \| **Class** \| **Tree** \| **Deprecated** \| **Index** \| **Help** \| **Weka's home** | |  |
| **PREV CLASS**   **NEXT CLASS** | **FRAMES**    **NO FRAMES**     **All Classes** |  |
| SUMMARY: NESTED \| FIELD \| CONSTR \| METHOD | DETAIL: FIELD \| CONSTR \| METHOD |  |


---
